# Risk of anaemia among women engaged in biomass-based fish smoking as their primary livelihood in the central region of Ghana: a comparative cross-sectional study

**DOI:** 10.1186/s40795-021-00456-w

**Published:** 2021-09-06

**Authors:** Daniel Armo-Annor, Esi K. Colecraft, Seth Adu-Afarwuah, Aaron Kobina Christian, Andrew D. Jones

**Affiliations:** 1 Department of Nutrition and Food Science, University of Ghana, P.O. Box LG 134, Accra, Ghana; 2 Regional Institute for Population Studies, University of Ghana, P.O. Box LG 96, Accra, Ghana; 3 School of Public Health, University of Michigan, Ann Arbor, MI 48109-2029 USA

**Keywords:** Anaemia, Fish smoking, Livelihood, Biomass fuel, Ghana

## Abstract

**Background:**

Fish smoking using biomass fuel is an important livelihood for women living in the coastal regions of Ghana and may contribute to anaemia risk. We assessed whether women who smoke fish as their primary livelihood are at increased risk of anaemia compared to women in other livelihoods in the Central Region of Ghana.

**Methods:**

We conducted a comparative cross-sectional study of 330 randomly selected adult women (18–49 years) whose primary livelihood was either fish smoking (FSL) involving the burning of biomass fuel (*n* = 175) or other livelihoods (OL) not involving burning of firewood (*n* = 155). Data on participants’ recent diet were collected from a single, quantitative 24-h dietary recall and qualitative 7-day food frequency questionnaire of animal-source food (ASF) consumption. We further assessed participants’ haemoglobin concentration using the Urit 12 Hemocue system. We compared total iron intakes, the proportion of dietary iron from animal and plant sources, mean haemoglobin concentrations, and anaemia prevalence between FSL and OL women.

**Results:**

Fish was the most frequently consumed ASF by both groups of women. Although OL women consumed more diverse ASFs in the past week compared with the FSL women (3.4 ± 1.2 vs. 2.7 ± 1.3; *p* < 0.001), the contribution of ASFs to total iron intake in the past day was greater for the FSL women (49.5% vs. 44.0%; *p* = 0.030). Estimated total dietary iron intake in the past day was generally low (5.2 ± 4.7 mg) and did not differ by group. The unadjusted prevalence of anaemia was 32 and 27.1% among the FSL and OL women, respectively (*p* = 0.33). After covariates adjustment, the FSL women had statistically higher anaemia prevalence (36.4% vs. 20.5%; *p* = 0.032) and 80% greater risk of being anemic (RR: 1.8; 95% CI: 1.1, 3.0) than the OL women.

**Conclusion:**

Women who use biomass fuel to smoke fish as their primary livelihood had an increased risk of anaemia. Furthermore, the average 24-h dietary iron intake among both the FSL and OL women was below their daily iron requirement. Interventions to enhance women’s dietary iron intake and reduce their livelihood related biomass smoke exposure may be warranted in this population.

**Supplementary Information:**

The online version contains supplementary material available at 10.1186/s40795-021-00456-w.

## Background

Anaemia prevalence among Ghanaian women of reproductive age (WRA) remains unacceptably high at 42% [1]. Anaemia has multiple etiologies including iron deficiency due primarily to suboptimal dietary iron intake, infections, worm infestations, and inherited blood disorders [2, 3]. The relative contribution of the various causes to the total burden of anaemia varies by region, population group and environmental factors [3]. According to the World Health Organization [4], about 50% of all anaemia is due to iron deficiency associated primarily with inadequate iron intake. However, in a systematic analysis of national surveys, the authors reported that countries with very high burden of inflammation (determined by an index derived from infection, hygiene, and/or overnutrition indicators) had the lowest prevalence of anaemia among WRA [5]. Furthermore, anaemia attributable to iron deficiency among WRA was considerably lower (about 16%) for countries with a severe public health burden of anaemia (i.e. prevalence greater than 40%) [5].

Smoke from burning biomass fuel is another source of inflammation which has also been implicated as a possible cause of anaemia in a few studies with children and women. Anaemia associated with biomass smoke is believed to stem from altered haemoglobin metabolism and disruption of red bloods cells resulting from systemic inflammation and oxidative stress respectively, induced by pollutants in the smoke [6–9]. Kyu et al. [10] conducted a multilevel analysis of data from Demographic and Health Surveys for 29 countries and found that country-level exposures to biofuel smoke were associated with up to a four-fold increased odds of anaemia among children under five years of age [10]. Using data from the 1998–1999 National Family Health Survey in India, Mishra & Retherford [11] reported a significantly higher risk of moderate-to-severe anaemia among preschool-aged children living in households where biofuels were used for cooking compared to those in households using cleaner fuels [11]. However, Machisa et al. [12] did not find a significant association between household use of biomass fuel and anaemia among preschool-aged children in Swaziland [12]. In India, pregnant women in the Nagpur district whose main source of fuel for cooking was biomass-based (i.e., wood, straw/shrubs/grass, agricultural crop, or animal dung) had a higher adjusted relative risk of both mild and moderate-to-severe anaemia [13]. Similarly, Sukhsohale et al. [14] found that among non-pregnant women from the same Indian district, those with the highest biomass smoke exposure index were significantly more likely to have anaemia [14]. In contrast, blood haemoglobin concentration did not differ between non-pregnant Guatemalan women who cooked with biomass-based smoke ovens versus those who used a smokeless stove [15].

Preserving fish using smoke (most commonly from burning wood) is widespread among approximately 185 fishing villages along Ghana’s coast and inland fishing communities where women make up about 70% of the workforce in the post-harvest fisheries value chain [16]. Indeed, fish smoking using firewood is the main livelihood of the majority of Ghanaian women living in coastal communities. Depending on the species and desired dryness, fish may be smoked for 2 to 18 h using smoking ovens that emit considerable amounts of smoke [17]. Thus, it is probable that being engaged in fish smoking as a livelihood may expose women to chronic smoke inhalation during the fish smoking process which may increase their risk of anaemia [18]. Furthermore, women who smoke fish as their primary livelihood may consume less diverse animal-source foods (ASF) because of greater dependence on fish and so limit their intake of comparatively richer sources of iron such as livestock meats. Kawarazuka and Bene [19] observed that due to variability in the iron content of different fish species, heavy dependence on fish at the expense of other ASFs may influence dietary iron intakes and predispose women to iron deficiency anaemia.

The Central Region of Ghana, the site of this study, is among the regions of Ghana with the highest prevalence of anaemia, and a large proportion of women in the area derive their livelihoods from fish smoking activities [1, 17]. It is unclear the extent to which women fish smokers consume fish and/or other ASFs and whether these women have a greater risk of anaemia. The aims of this comparative cross-sectional study were to compare among women whose primary livelihood is fish smoking and those engaged in other livelihoods not involving the burning of firewood: i) mean haemoglobin concentration and prevalence of anaemia, and ii) animal-source food intakes and their contribution to total iron intakes. We hypothesized that women engaged in fish smoking as their primary livelihood would have a greater risk of anaemia possibly due to higher smoke exposure and/or consumption of less diverse ASFs.

Findings from this study will add to the body of evidence on the association between biomass fuel use and anaemia among WRA and delineate whether there are unique considerations needed in efforts to address anaemia in WRA whose primary livelihoods expose them to chronic biomass smoke inhalation.

## Methods

### Study design, setting and participants

This comparative cross-sectional study was carried out in Biriwa, a fishing community of 7086 people in the Mfantseman Municipality of the Central Region of Ghana [20]. Given its relatively large size, Biriwa was purposively selected from six fishing villages that were part of a larger pilot study conducted in the Central Region (the Invisible Fishers study) from May 2018 to August 2019 examining the impacts of various fisheries value chain and other behavior change interventions on anaemia among women of reproductive age (WRA) [21].

The inclusion criteria for participation in the study were, being an adult non-pregnant non-lactating WRA (18 to 49 years), living in Biriwa and being willing to participate in the study. A screening census was completed to list all adult women in the community according to their primary livelihood for the past two years; whether fish smoking involving burning of firewood (FSL women) or other livelihoods not involving burning of firewood and living in a household where no one smokes fish as a major economic activity (OL women). Due to concern about not achieving the estimated sample size, no restrictions were placed on the number of women who could be listed per household during the census. A total of 355 eligible FSL and OL women in 311 households were listed. Women participating in the Invisible Fishers study in Biriwa (*N* = 10) were ineligible to participate in this study. A sample size of 175 participants per group (i.e., FSL and OL) was estimated based on a 5% level of significance, 80% power, expected anaemia prevalence of 50% (using baseline anaemia prevalence for the Central Region from the Invisible Fishers study) and 35% (using prevalence of anaemia in the Upper West Region of Ghana) where fish smoking is expected to be only minimally practiced [1] among the FSL women and OL women, respectively, and 3% contingency to cater for incomplete surveys. As 194 eligible FSL women were listed from the census, we randomly selected 175 to participate in the study using the RAND function in excel. About 30% (*n* = 52) where from the same households (two or three [two instances] women from the same household). The remaining 19 eligible women were put on a waiting list and replaced women who could not complete the study due to reasons such as refusals, travel and relocation. The number of eligible OL women listed (*n* = 161) was lower than the estimated sample size so all of them were invited to participate in the study. About 19% (*n* = 30) where from the same household (two or three [one instance] from the same household).

### Data collection and measurement of variables

Data collection took place from December 2018 to February 2019 and was completed with semi-structured questionnaires. Questionnaires were designed using the Kobotoolbox platform and loaded on Android tablets using the Open Data Kit (ODK) for data collection. Three research assistants with at least high school education were recruited and trained by the primary researcher to support the data collection activities. The questionnaire was pre-tested on women with similar characteristics in a neighboring community before it was administered to the actual study respondents. Face-to-face interviews were completed with participants in their preferred local language (Fante or Twi) or English at their homes or workplaces. Data were recorded with Android tablets by direct electronic data entry using the ODK.

The research assistants obtained information on household characteristics, as well as personal social demographic characteristics, reproductive history, health, recent diet and use of firewood from the selected participant in each household (see Additional file 1). Using the Urit12 HemoCue (URIT Medical Electronics Co., LTD, China) system, a lancet was used to prick the forefinger of each participant and a drop of blood was gently squeezed onto the sampling point of the system to obtain a digital reading of the haemoglobin concentration in the sample. Anaemia was defined as having haemoglobin concentration of less than 12 g/dl [22] based on one sample per participant. A one day 24-h recall method was used to record all foods and beverages (except water) consumed by the study participant in the past 24 h [23]. Wooden food models and household measures were used to help participants estimate quantities consumed. The frequency of consumption of different ASFs by the participant in the past week was captured with an abbreviated food frequency questionnaire listing seven categories of commonly consumed ASFs including fish and seafood, milk and milk products, livestock meats, eggs, poultry, organ meats and bush meats (see Additional file 1). This was a semi-quantitative questionnaire which required participants to specify the number of days in the past week they ate a particular ASF without specifying the portion size. From the 24-h recall data, we determined each participant’s dietary diversity score based on the 10 food groups used to compute the FAO’s Minimum Dietary Diversity for Women indicator [24] and computed recent total iron intakes for participants. The iron content of foods consumed in the past 24-h was determined using a food database (RIING food composition database, Nutrition Department, University of Ghana, unpublished). We estimated the bioavailability of the iron from the foods consumed using a previously published method [25]. For each eating event, 40% of the iron content of meat, fish and poultry (MFP) consumed was considered as heme iron and available and the remaining 60% non-heme while 100% of iron from non-animal sources consumed was considered non-heme. Bioavailability of non-heme iron was computed as 5, 10% or 15% of the total iron content of the food source depending on the quantities of MFP and vitamin C consumed in the same eating event or meal [25]. The proportion of the total estimated bioavailable iron intake from all foods contributed by ASFs consumed in the 24-h recall period was computed. Additionally, we calculated ASF diversity as the number of different categories of ASF (out of a total of seven) consumed by the participant in the past seven days. Completed questionnaires were reviewed for completeness at the end of each day. Participants with missing or incomplete responses were contacted the following day to complete the missing information.

### Data analysis

Data were managed, cleaned and analyzed using the Statistical Software Package for Social Sciences (SPSS) version 22.0 (Chicago, USA) and SAS for Windows Release 9.4 (Cary, NC, USA). One duplicate record was identified and removed during data cleaning, otherwise all completed questionnaires were included in the analysis. Bivariate analyses using Student’s T-tests for continuous variables and Chi-Square tests for categorical variables were used to summarize differences in background and household characteristics of the FSL and OL women. Additionally, we compared group differences in mean ASF diversity (number of different ASFs consumed in the past seven days), total iron intakes, and mean percent contribution of ASFs to total iron intakes in the past 24-h.

Blood haemoglobin concentrations were compared between the two groups of women using a general linear model and ANCOVA for unadjusted and adjusted comparisons, respectively. The SAS PROC GLIMMIX procedure was used in both cases. Unadjusted and adjusted means with their 95% CIs were calculated. Anaemia prevalence was compared using a simple logistic regression model for an unadjusted comparison, and multiple logistic regression model for an adjusted comparison. The SAS PROC GLIMMIX procedure was used in both cases. A binary distribution and log-link function were specified in the SAS procedures so that relative risks between groups and their 95% CIs were calculated. Covariates for the ANCOVA and logistic regression models were selected by correlating anaemia with each covariate so that only those independent variables significantly associated with the outcome at alpha = 0.2 [26] were selected for the multiple logistic regression model. This conservative level of alpha was chosen to minimize the risk of type II error in variable selection [27]. In addition to covariates selected through correlation analysis (i.e., marital status, fever in the past two weeks, fish smoking livelihood, dewormed in past 3 months, ever been pregnant and age), other covariates including number of days spent smoking fish, fuel for cooking, ASF diversity, and access to a toilet facility were selected for the final ANCOVA and multiple logistic regression models if they were associated with haemoglobin concentration or anaemia, respectively.

## Results

### Background and household characteristics of women

A total of 355 women were invited to participate in the study and 330 (93%) of them completed the survey. Out of the 25 (7%) who did not participate, 12 (48%) refused to participate (FSL women = 10, OL women = 2), 11 (44%) were traveling out of the study area at the time of the survey (FSL women = 7, OL women = 4) and 2 (8%) FSL women could not be located on the scheduled day or after two additional attempts to interview them. Of the 330 women who completed the study, 175 (53%) were FSL women and 155 (47%) were OL women. The OL women were largely engaged in occupations such as hairdressing, dressmaking, petty trading, fish mongering, farming, and cleaning services as their primary livelihoods, with < 1% employed in professional (nursing and teaching) occupations. The FSL women smoked fish on average 4.9 ± 1.3 times a week for a mean of 3.9 ± 1.7 h per day. With the dominance of fish smoking in the community 59% (*n* = 95) of the OL women indicated that they occasionally assisted relatives or neighbours with fish smoking for an average of less than twice per week. Only two OL women reported having the sickle cell trait.

Background characteristics of the two groups of women in the study are summarized in Table 1. Compared to OL women, the FSL women were on average older (38 vs 29 years; *p* < 0.001), more likely to be married (82% vs. 57%; p < 0.001) and to have no formal education (88% vs. 50%; p < 0.001). The FSL women also had smoked fish as their primary livelihood for twice the number of years that the OL women had spent in their primary livelihoods. The OL women were less likely than the FSL women to have a supplementary income source and significantly more of them reported that they earned less than 500 Ghana Cedis (about 100 US dollars) per month. There were no group differences in the proportion of women who had taken deworming medication or an iron supplement in the past three or six months, respectively. The FSL women were more likely to report usually sleeping under a mosquito net. There were no group differences in the use of firewood for cooking or in living with someone who smokes cigarettes in the household.

**Table 1:** Background characteristics of women in fish smoking livelihoods (FSL) or other livelihoods (OL) in the study

Characteristic	FSL women (***n*** = 175)	OL women (***n*** = 155)	^a^P-value
Age, years	38 ± 8^b^	29 ± 8	< 0.001
Married	143 (82)^c^	89 (57)	< 0.001
No Formal education	87 (50)	18 (12)	< 0.001
Years in primary occupation	12 ± 7	6 ± 5	< 0.001
Has supplementary income source	78 (45)	45 (29)	< 0.001
Average monthly income < GH₵ 500^d^	103 (59)	130 (84)	< 0.001
Has been pregnant before	170 (97)	107 (69)	< 0.001
Parity	5 ± 3	2 ± 2	< 0.001
Dewormed in past 3 months	47^c^ (27)	47 (30)	0.50
Used iron supplements in past 6 months	25 (14)	27 (17)	0.44
Usually sleeps under mosquito net	106 (61)	72 (47)	< 0.001
Had fever in past 2 weeks	47 (27)	29 (19)	0.08
Household size	6 ± 3	5 ± 3	< 0.001
Household has improved toilet facility	44 (25)	36 (41)	< 0.001
Use firewood as main fuel for cooking	28 (16)	17 (11)	0.18
Cigarette smoker in household	9 (5)	5 (3)	0.39

^a^Significance associated with independent T-test for continuous variables, and chi-squared statistic for categorical variables ^b^Mean ± SD; ^c^n (%); ^d^exchange rate at time of the study I USD = 5GHC

### Recent diet, animal-source food diversity and iron intakes among women

**Fig. 1:**
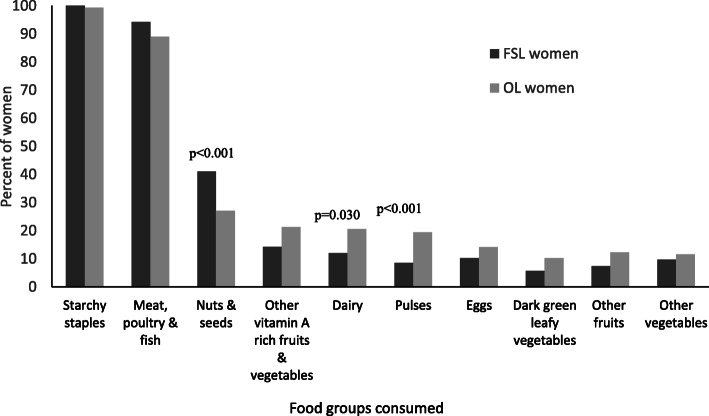
Food groups consumed by women in the study in the past 24 h

**Fig. 2:**
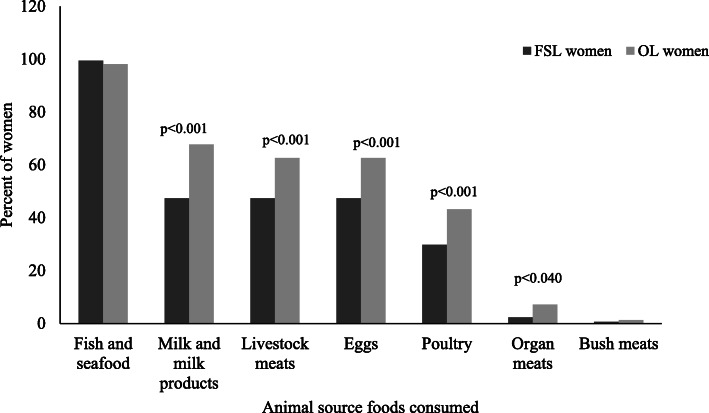
Types of Animal Source Foods (ASF) consumed in the past 7-days by women in the study

Starchy staples (including grains, white roots and tubers, and plantains) were consumed by almost all the women in the past day and at least 89% of women consumed meat, poultry, and fish in the past day **(**Fig. 1**).** Less than 50% of women in both groups consumed foods from the other eight food groupings. Consumption prevalence for the different food groups was similar in the two groups of women except for nuts and seeds where consumption prevalence was significantly higher among FSL women and significantly more OL women consumed foods from the dairy and pulses food groups. In the past day, the total iron intake for both groups of women from both plant and animal sources was about 5 mg (Table 2). Mean percent contribution of iron from ASFs was, however, significantly higher for the FSL women (49.5% vs. 44.0%; *p* = 0.030). Differences in the types of ASFs consumed by the women in the past week are depicted in Fig. 2. Fish and seafood were the predominant ASF consumed by both groups of women in the past week. Less than 50% of FSL women consumed other types of ASF in the past week, whereas at least 60% of the OL women consumed foods made with dairy, livestock meats and eggs in the past week in addition to consuming foods from the fish and seafood group. Consumption of poultry and organ meats was also higher among OL women. Organ meats and bush meats (game) were only minimally consumed. Mean ASF diversity was significantly higher among the OL women (3.4 ± 1.2 vs. 2.7 ± 1.3; *p* < 0.001). However, with the exception of fish and seafood where the mean frequency of consumption in the last week was at least 12 times, the mean frequency of consuming the other ASF groups averaged about two or fewer times in the past week (Table 3).

**Table 2:** Quantities of Animal Source Foods (ASF), total iron intake and contribution of ASF to total iron intakes in the past day

Variable	^a^FSL women (n = 175)	^b^OL women (n = 155)	Mann-Whitney U	^c^P-value
Fish and shell fishes (g)	90.8^d^	78.0	10,077.5	0.68
Livestock meat (g)	11.0	19.0	404.0	0.15
Milk and milk products (g)	30.0	32.0	376.0	0.67
Eggs (g)	124.4	100.0	172.0	0.15
Poultry (g)	78.0	78.0	110.5	0.88
Total iron intake from all sources (mg)	5.3 ± 5.2^e^	5.0 ± 4.2	–	0.57
Mean percent contribution of iron from ASF	49.5 ± 22.2	44.0 ± 23.9	–	0.030

^a^FSL = Fish Smoking Livelihood ^b^OL = Other Livelihood ^c^P-value associated with Mann-Whitney U test or independent T-test, ^d^Median, ^e^Mean ± SD

**Table 3:** Differences in mean ± SD frequency of consuming different Animal Source Foods (ASF) in the past week

ASF group	FSL^a^ women (n = 175)	OL^b^ women (n = 155)	^c^p-value
Fish and seafood	14.2 ± 3.8	12.4 ± 4.7	< 0.001
Milk and milk products	1.2 ± 2.2	1.9 ± 2.1	< 0.001
Livestock meats	1.1 ± 1.5	1.3 ± 1.6	0.09
Eggs	0.9 ± 1.4	1.2 ± 1.4	0.09
Poultry	0.5 ± 0.9	0.9 ± 1.5	< 0.001
Organ meats	< 0.1 ± 0.2	0.1 ± 0.3	0.09
Bush meats	< 0.1 ± 0.1	0.1 ± 0.2	0.36

^a^Fish Smoking Livelihood ^b^Other Livelihoods ^c^*P*-value associated independent T-test

### Unadjusted and adjusted differences in haemoglobin concentration and anaemia prevalence between FSL women and OL women

**Fig. 3:**
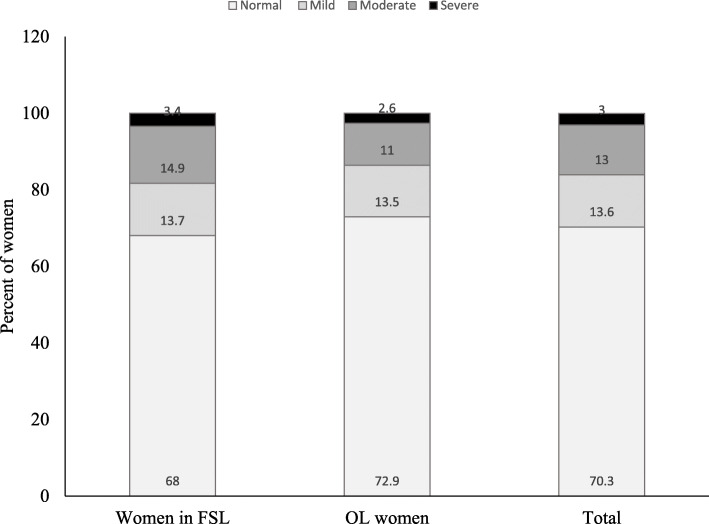
Prevalence of anaemia among women in fish smoking livelihoods and other livelihoods in the study

In unadjusted analyses, mean ± SD haemoglobin concentration did not differ between the two groups of women (FSL, 12.3 ± 1.9 g/dl; OL, 12.6 ± 1.8 g/dl; *p* = 0.17). About one-third of all the study women had mild (13.6%), moderate (13%) or severe (3%) anaemia (Fig. 3) and there was no group difference in the prevalence of any anaemia among the women (FSL, 32%; OL, 27%; *p* = 0.33). After controlling for covariates, livelihood type was associated with haemoglobin concentration and the prevalence of anaemia. That is, the FSL women had significantly lower mean ± SD haemoglobin concentration (12.2 ± 0.2 g/dl vs. 12.8 ± 0.2 g/dl; *p* = 0.018) (Table 4), and higher prevalence of anaemia (36.4% vs. 20.5%; *p* = 0.032) than the OL women. The risk of anaemia was 80% greater in the FSL women compared with the OL women (Table 5).

**Table 4:** Unadjusted and Adjusted differences in mean haemoglobin concentration between women in the study

	FSL women^a^ (n = 175)	OL women^b^ (n = 155)	Difference in means (95% CI)^c^	^d^P-value
**Haemoglobin concentration (g/dL)**				
Unadjusted	12.3 ± 1.9	12.6 ± 1.8	−0.3 (−0.7, 0.1)	0.17
Adjusted^e^	12.2 ± 0.2	12.8 ± 0.2	−0.6 (−1.2, − 0.1)	**0.018**

^a^ Women engaged in Fish smoking livelihood. ^b^ Women engaged in other livelihoods unrelated to fish smoking ^c^95% CI = 95% Confidence Interval ^d^P-values are based on general linear regression model (unadjusted) and ANCOVA (adjusted) for haemoglobin concentration ^e^Adjusted for marital status, toilet facility, fever, fuel used for cooking, cigarette smoker in household, exposed to cigarette smoke outside the home at least once in past 12 months, number of days spent smoking fish in a week, and Animal Source Food (ASF) diversity in past 7-days

**Table 5:** Unadjusted and adjusted odds ratios for having anaemia among women in the study

Variable (referent)	Unadjusted		Adjusted	
	Relative Risk (95% Cl)^b^	^a^P-value	Relative Risk (95% Cl)^b^	^a^P-value
**Livelihood type**				
FSL (OL)^c^	1.2 (0.8, 1.7)	0.331	1.8 (1.1, 3.0)	**0.032**
**Age**				
**< 35 years (**≥35 years)			1.3 (0.9, 2.0)	0.17
**Marital status**				
Married (Single)			0.9 (0.6, 1.3)	0.55
**Ever been pregnant**				
Yes (No)			0.8 (0.4, 1.3)	0.33
**Dewormed in past 3mos**				
**No (**Yes)			0.8 (0.6, 1.2)	0.30
**Fever in past 2 weeks**				
Yes (No)			1.2 (0.8, 1.7)	0.47
**Household with toilet facility**				
No (Yes)			0.7 (0.5, 1.1)	0.09
**Cooking fuel**				
Biomass fuel (other fuel)			1.2 (0.8, 2.0)	0.38
**number of days spent smoking fish**				
More days (Fewer days)			0.7 (0.5, 1.2)	0.20
**ASF diversity**				
Low (High)			1.4 (1.0, 2.1)	0.08
**% of iron from ASF**				
Low (High)			1.2 (0.9, 1.7)	0.22

^a^ P-value is based on logistic regression: Dependent variable = Anaemia (Present/Not Present)

^b^ 95% CI = 95% Confidence Interval ^c^Fish smoking Livelihood (Other Livelihoods)

## Discussion

The study examined whether being engaged in fish smoking as a primary livelihood is associated with a higher risk of having anaemia among Ghanaian women compared to being engaged in a primary livelihood that does not involve burning of biomass smoke. Regardless of livelihood type, anaemia prevalence among women in the study was high although lower than the national average for rural communities (43%) and for the Central Region of Ghana (48%) [1]. The results show that women who burn biomass fuel to smoke fish as their primary livelihood had lower blood haemoglobin concentrations and a higher burden of anaemia than those whose primary livelihoods did not involve burning of biofuels. Our findings corroborate those from several previous epidemiological studies that have reported an association between use of biomass fuel and increased likelihood of anaemia among preschool-aged children and women [10, 11, 13, 14, 28]. The biological mechanisms by which exposure to biomass fuel lead to lower haemoglobin concentrations and higher anaemia risk are believed to involve cytokine-mediated inflammation in response to pollutants in biomass smoke such as carbon monoxide, transition metals, and particulate matter less than 10 μm (PM_10_) and less than 2.5 μm (PM_2.5_) in diameter [13, 29]. Anaemia caused by systemic inflammation impairs iron homeostasis and red blood cell synthesis leading to low serum iron levels similar to iron-deficiency anaemia, but unlike iron-deficiency, does not depress iron stores [30].

In previous studies linking anaemia with smoke exposure, the source of smoke exposure was household use of biomass fuel for cooking indoors. However, in the present study the prevalence of using biomass fuel for household cooking was similar for the two groups of women and was not significantly associated with anaemia prevalence. In the study community, fish smoking is largely done outdoors although some of the women smoked fish in semi-enclosed structures. Our results suggest that exposure to outdoor smoke from fish smoking among the FSL women might have had a similar impact on haemoglobin concentration and anaemia prevalence as did exposure to indoor biomass fuel in those previous studies. In fact outdoor exposure has also been shown to be associated with anaemia in children. For example, Morales-Ancajima et al. [31], observed that Peruvian children living in areas of Lima with higher outdoor concentrations of the biomass smoke pollutant particulate matter (PM_2.5_) had lower mean haemoglobin concentrations and higher anaemia prevalence than those living in areas with lower concentrations of the pollutant [31]. Honda et al. [32] reported that PM_2.5_ was responsible for a 0.81 g/dL decrease in average haemoglobin among old American adults [32].

Spending more days smoking fish was not associated with a higher risk of anaemia, though after controlling for covariates, a fish smoking livelihood was associated with a lower haemoglobin concentration and anaemia prevalence. Number of days of fish smoking may not necessarily reflect intensity of smoke exposure experienced by women as, depending on fish species and the type and stage of fish smoking, the number of actual hours spent near the smoking stove may differ widely [17]. Furthermore, there may have been limited variability in the women’s fish smoking enterprises thus limiting differences in the types of fish being smoked and in smoking method. Alternatively, the lack of association between number of fish smoking days and anaemia may mean that the differences observed by livelihood type may not be due entirely to smoke exposure from smoking fish. Given the multiplicity of anaemia causes even within individuals, one risk factor such as smoke exposure is unlikely to explain the total burden of anaemia in an individual or population [33]. In addition to biomass smoke exposure being a potential cause of anaemia, anaemia may be caused by various factors associated with nutritional deficiencies, infection and infestations and genetic haemoglobin disorders [34]. Hence, being engaged in fish smoking as a primary livelihood may predispose women to other anaemia risk factors that were not measured in this study. The use of biomass fuel has been linked to certain infectious diseases such as tuberculosis that are also associated with increased risk of anaemia [13, 35].

Fish was the predominant ASF in the diets of both groups of women. This was expected as fish is the cheapest animal protein source across Ghana and contributes to more than 50% of total animal protein intake [36, 37]. The two groups of women had similar 24-h total iron intakes from all sources and the contribution of iron from ASF was higher for the FSL women who had higher mean frequency of fish consumption in the past week. While the prevalence of consuming other ASF besides fish was higher for OL women, mean consumption frequency for iron-rich ASF such as livestock meats, organ meats, and poultry was low and not significantly different between the two groups. This suggests that fish was the main contributor to total iron intakes for both groups of women. This is plausible as the fish commonly available in the study community were the smaller fish species such as anchovy, sardinella, and herrings that tend to be richer sources of iron than the larger species because they are eaten whole with bones, head and viscera which have the highest concentration of iron and other micronutrients [19]. Cooking methods may also affect the iron content of animal protein sources. For example, Pourkhalili et al. [38] reported higher iron retention in grilled lamb meat compared to when the meat is boiled [38]. Total iron intake for both groups of women in the past day was just around 5 mg which falls substantially short of the recommended daily intake of 19.6 for adult women assuming dietary iron bioavailability of 15%. At even lower dietary bioavailability, levels expected for predominantly plant-based diets, the recommended daily intakes are even higher (62 mg, 31 mg, and 25.8 mg for 5, 10 and 12% bioavailability, respectively) [39]. In addition to smoke exposure, nutritional iron inadequacy is probably an important contributor to anaemia in the study population. Coexistence of iron-deficiency anaemia and anaemia of inflammation from infections is reportedly common among developing country populations [40, 41]. Efforts to enhance the women’s dietary diversity and consumption of iron-rich foods are also warranted in the community.

### Limitations

Our study has some limitations. First the two groups of women were from the same community where fish smoking is the dominant livelihood for women so that we did not have a true ‘unexposed’ group because: 1) more than 50% of women classified as being in primary livelihoods unrelated to fish smoking occasionally assisted with smoking activities of families and friends; and, 2) fish smoking was generally an outdoor activity indicating community-wide exposure to smoke. This may have underestimated the effect observed. Further, smoke exposure was not directly measured and so we cannot be truly certain whether the association observed was due to smoke exposure per se or other unmeasured characteristics that the women had. Iron intake was based on one 24-h recall which may not necessarily reflect usual intakes among the participants.

## Conclusion

The adverse effects of biomass smoke exposure on morbidity and mortality associated with respiratory and cardiovascular conditions and cancers in developing country populations has been long recognized, however the association with anaemia has received less attention. Our study suggests that women who smoke fish as their primary livelihood may have additional risks of anaemia besides those posed by other well recognized contributors to anaemia such as poor diet, and infections. This added risk may be especially important for reducing population-level anaemia risk given how ubiquitous fish smoking activities are across Ghana and throughout many communities in West Africa as described previously. The study also showed that the women’s iron intake in the past 24-h was well below the required iron intake with probability the women had underlying iron deficiency. Therefore, efforts to reduce smoke emissions from fish smoking ovens and to enhance dietary iron intake may be warranted in efforts to address different causes of anaemia in our study community and beyond given the potential for impact at scale. Further research is needed to understand the mechanisms of the association and to determine causality.

## Supplementary Information

**Additional file 1.** Questionnaire on the risk of anaemia among women engaged in biomass-based fish smoking as their primary livelihood in the Central Region of Ghana. The word file contains the questionnaire used to gather information on the personal social demographic characteristics, reproductive history, health and use of firewood of the study participants and another section showing the modified food frequency questionnaire.

## Data Availability

The datasets used or analysed during the current study are available from the corresponding author on reasonable request.

## Abbreviations

ASF: Animal Source Food
FAO: Food and Agriculture Organization
FSL: Fish Smoking Livelihood
OL: Other Livelihood PM: Particulate Matter
WHO: World Health Organization
WRA: Women of Reproductive Age
