# Supplementary material for: Risk of anaemia among women engaged in biomass-based fish smoking as their primary livelihood in the central region of Ghana: a comparative cross-sectional study
Source: BMC Nutr. 2021 Sep 6;7:50. doi: 10.1186/s40795-021-00456-w (PMC8420040; doi:10.1186/s40795-021-00456-w)
Supplement: Supplementary file 1 — Additional file 1. Questionnaire on the risk of anaemia among women engaged in biomass-based fish smoking as their primary livelihood in the Central Region of Ghana. The word file contains the questionnaire used to gather information on the personal social demographic characteristics, reproductive history, health and use of firewood of the study participants and another section showing the modified food frequency questionnaire. [file 40795_2021_456_MOESM1_ESM.docx]

**QUESTIONNAIRE ON THE RISK OF ANAEMIA AMONG WOMEN ENGAGED IN BIOMASS-BASED FISH SMOKING AS THEIR PRIMARY LIVELIHOOD IN THE CENTRAL REGION OF GHANA: A COMPARATIVE CROSS-SECTIONAL STUDY**

Interviewer’s name: ______________________ Interview date: __________

Household No.: __________ Participant ID: ________

**SECTION A**

| **PART A: BACKGROUND INFORMATION**  ***I would start by asking you some personal questions about yourself*** | | |
| --- | --- | --- |
| ***Socio-demographic Characteristics*** | | |
| 1 | How old are you? | _________ years |
| 2 | How many years have you lived in this community? | __________ years |
| 3 | What is your ethnic background? | 1= Akan  2= Ga/Adangbe  3= Ewe  4= Northern ethnicity  5= Other (specify) |
| 4 | What is your religion? | 1= Christian  2= Muslim  3= Traditional religion  4 = Other (specify) |
| 5 | What is your current marital status | 1= Single/Never been married  2= Married  3= Divorced  4= Widowed |
| 6 | What is the highest level of formal education you have completed? | 1=< Primary  2= Primary  3= JHS  4= SHS  5= Vocational training  6= Tertiary |
| 7 | What is the main work you do for income? | 1= Fish smoking  2= Other fish related activity  3= Farming  4= Trading  5= Vocational  6= Not working  7= Other (specify) |
| 8 | How long have you been in this line of work? | _________ years |
| 9 | Do you have a supplementary source of income? | 1= Yes  2= No |
| 10 | If yes, what is the supplementary work you do for income? | 1= Fish smoking  2= Other fish related activity  3= Farming  4= Trading  5= Vocational  6= Other (specify) |
| 11 | During this time of the year, how much do you earn monthly? | 1=<500 GH₵  2= 500-999 GH₵  3= 1000-1999 GH₵  4= 2000-3999  5= 3000-4999 GH₵  6=≥5000 GH₵ |
| ***Household Characteristics***  ***I would like to ask you a few questions about your household*** | | |
| 12 | Who is the head of this household? | 1= Myself  2= Husband  2= Father or father in-law  3= Mother or mother in-law  4= Other male household member  5= Other female household member |
| 13 | How many people in total are part of your household? | **_______** |
| 14 | How many of your household members are children  (<18 years)? | **_______** |
| 15 | How many of your household members are adults  (≥ 18 years) | **_______** |
| 16 | What is your household’s main source of drinking water? | 1= Pipe  2= borehole  3= Well  4= River/stream  5= Rainwater  6= Sachet water  7= Other (specify) |
| 17 | How do you treat your household’s water before drinking? | 1= No treatment  2= Boil  3= Filter  4= Chlorine/bleach  5= Let it stand and settle  6= Other (specify) |
| 18 | What kind of toilet facility does your household use? | 1= Water closet (WC)  2= Public toilet/KVIP  3= Nearby (Bush/beach/field)  4= Pit latrine with slab  5= Pit latrine without slab  5= Other specify |
| 19 | Do you share your household’s toilet facility with other members who are not part of your household? | 1=Yes  2=No |
| 20 | What is your main source of light? | 1= Electricity  2= Kerosene lamp  3= Candle  4= Solar lamp  5= Other (specify) |
| 21 | Does your household have any of the following assets? | 1= Electricity  2= Radio  3= Black/white television  4= Color television  5= Mobile phone  6= Refrigerator  7= Generator  8= Video deck/DVD/VCD  9= Bed  10= Table  11= Access to internet in any  12= Motorcycle  13= Car or truck |
| 22 | Main material of the floor (sleeping place)  Record Observation | 1= Cement  2= Earth/sand  3= Ceramic/tiles/terrazzo  4= Carpet  5= Other (specify) |
| 23 | Main material of the roof.  Record Observation. | 1= Palm leaf  2= Palm/Bamboo  3= Wood planks  4= Roofing sheets  5= Ceramic/brick tiles  6= Cement  7= Other (specify) |
| 24 | Main material of the exterior walls  Record Observation. | 1= Bamboo with mud  2= Plywood  3= Stone with mud  4= Cement  5= Bricks  6= Other (specify) |
| **Now I would like to ask you some questions about cooking in your household and other household practices** | | |
| 25 | What is the main fuel used for cooking in your household? | 1= Electricity  2= LPG  3= Charcoal  4= Kerosene  5= Wood  6= Animal dung  7= No food cooked in household |
| 26 | Is the cooking usually done in the house, in a separate building, or outdoors? | 1= In the house  2= In a separate building  3= Outdoors |
| 27 | Who spends most of the time cooking for the household? | 1= Myself  2= Another household member  3= A non-household member |
| **PART C: HEALTH INFORMATION**  ***At this point I will ask questions about your health*** | | |
| 28 | Have you ever been pregnant? | 1= Yes  2= No |
| 29 | If yes, how many live births have you had? | ______ |
| 30 | Do you have sickle cell trait? | 1= Yes  2= No  3= Don’t know |
| 31 | Have you had fever in the last two weeks? | 1= Yes  2= No |
| 32 | If yes, what did you do to treat it? | 1= Hospital  2= Medicine from pharmacy  3= Herbal medicine  4= Nothing  5= Other (specify) |
| 33 | When was the last time you dewormed? | 1= Less than one month ago  2= Within 6 months  3= Less than a year ago  4= More than 2 years ago  5= Don’t know |
| 34 | Do you usually sleep under a mosquito net at night? | 1= Yes  2= No |
| 35 | Did you sleep under a mosquito net last night? | 1= Yes  2= No |
| 36 | Do you smoke cigarettes, cigars or tobacco? | 1= Yes  2= No |
| 37 | If yes, during the past 30 days, did you smoke cigarettes, cigars or tobacco? | 1= Yes  2= No |
| 38 | Does anyone in your household smoke? | 1= Yes  2= No |
| 39 | Have you taken iron supplements in the last 6 months? | 1= Yes  2= No |
| 40 | Over the past 12 months, how often have you gone to places other than your home or work where people smoked around you indoors, close enough to see or smell the smoke?  ***(Applicable to OL women)*** | 1= More than once a week  2= More than once a month  3= Less than once a month  4= Never |
| **PART D: USE OF BIOMASS FUEL**  ***Now I will ask you some questions about your exposure to smoke from biomass fuel*** | | |
| 41 | On an average, about how many times in a week do you help someone who smokes fish in her business during this season?  *Applicable to OL women only* | ______ days |
| 42 | During the past 12 months, about how many months did you participate in fish smoking?  *Q43 to Q47 Applicable to women in FSL only* | ______ months |
| 43 | On average, about how many times in a week do you smoke fish during this time of the year? | ______ days |
| 44 | On average, how many hours in a day do you spend smoking fish? | ­­  _________ Hours |
| 45 | What kind of smoke oven do you use? | 1= Chorkor  2= Ahotor  3= Round clay oven =  4= Metal drum =  5= Traditional kiln =  6= Frismo/Cosmos stove  7= FTT =  8= LPG Gas Burner =  9= Other (specify) |
| 46 | What is the main source of fuel you use for smoking fish? | 1= Firewood  2= Charcoal  3= Animal waste (cow dung)  4= Other (specify) |

**SECTION B**

**FOOD FREQUENCY**

*Next I would like to ask you some questions about intake of animal source foods you ate in the last seven days, since last* [SAY DAY, SAME AS INTERVIEW DAY]. *For each food I ask about, please tell me how many times in the last seven days you think you ate that food*

| **FOOD ITEMS** | Number of days |
| --- | --- |
| Livestock meats (All types of red meat e.g. goat, cow, lamb, pork) | ___________ |
| Bush meats (E.g. Grass cutter, antelope, rat, squirrel and other wild animals) | ___________ |
| Organ meats (Blood-based foods e.g. liver, kidney, heart, gizzard, lung etc.) | ____________ |
| Poultry (Chicken, guinea fowl, duck and other birds) | ___________ |
| Fish and shell fish (Fresh or dried fish e.g. Anchovies, one-man-thousand or other small fish eaten whole, either fresh or dried, shrimps/amonkor, adode, crabs) | ___________ |
| Milk and milk products (Milk, cheese, yogurt or other milk products) | ___________ |
| Eggs (Any type of egg e.g. chicken eggs, duck eggs etc.) | ___________ |
